# The Ventilation of the South-Eastern Hospital

**Published:** 1907-06-01

**Authors:** 


### THE VENTILATION OF THE SOUTH-EASTERN HOSPITAL.

Sir,—We notice in your issue of May 25 a paragraph to the effect that the South-Eastern Hospital, of which we are the architects, has been ventilated on the "Boyle" natural system. This is not the case; the system is purely mechanical by means of fans, and was designed by ourselves.

Yours truly,

May 27, 1907. Thos. W. Aldwinckle & Son.

[We published the statement as we received it from Messrs. Robert Boyle and Son, 64 Holborn Viaduct, London, who now write, under date May 29, that 32 of their patent air-pump ventilators were supplied and fixed on the external brick upcast shafts in connection with the wards of the latest additions to the South-Eastern Hospital.—Ed.]

